# Regression Analysis of Orthogonal, Cylindrical and Multivariable Color Parameters for Colorimetric Surface pH Measurement of Materials

**DOI:** 10.3390/molecules26123682

**Published:** 2021-06-16

**Authors:** Katarína Vizárová, Izabela Vajová, Naďa Krivoňáková, Radko Tiňo, Zdenko Takáč, Štefan Vodný, Svetozár Katuščák

**Affiliations:** 1 Department of Wood, Pulp and Paper, Institute of Natural and Synthetic Polymers, Faculty of Chemical and Food Technology, Slovak University of Technology in Bratislava, Radlinského 9, 812 37 Bratislava, Slovakia; izabela.vajova@stuba.sk (I.V.); radko.tino@stuba.sk (R.T.); svetozar.katuscak@stuba.sk (S.K.); 2 Institute of Information Engineering, Automation, and Mathematics, Faculty of Chemical and Food Technology, Slovak University of Technology in Bratislava, Radlinského 9, 812 37 Bratislava, Slovakia; nada.krivonakova@stuba.sk (N.K.); zdenko.takac@stuba.sk (Z.T.); 3 Certex a.s., Radlinského 9, 812 37 Bratislava, Slovakia; stefan.vodny@gmail.com

**Keywords:** pH measurement, colorimetric pH-metry, acidity, acid paper, deacidification

## Abstract

The surface pH is a critical factor in the quality and longevity of materials and products. Traditional fast colorimetric pH detection-based tests such as water quality control or pregnancy tests, when results are determined by the naked eye, cannot provide quantitative values. Using standard pH papers, paper-printed comparison charts, or colorimetric microfluidic paper-based analytical devices is not suitable for such technological applications and quality management systems (QMSs) where the particular tested material should contain a suitable indicator in situ, in its structure, either before or after the process, the technology or the apparatus that are being tested. This paper describes a method based on the combination of impregnation of a tested material with a pH indicator in situ, its exposure to a process of technology whose impact on pH value is to be tested, colorimetric pH measurement, and approximation of pH value using derived pH characteristic parameters (pH-CPs) based on CIE orthogonal and cylindrical color variables. The hypotheses were experimentally verified using the methyl red pH indicator, impregnating the acid lignin-containing paper, and preparing a calibration sample set with pH in the range 4 to 12 using controlled alkalization. Based on the performed measurements and statistical evaluation, it can be concluded that the best pH-CPs with the highest regression parameters for pH are *√*∆*E, ln (a),* *√*∆*H (ab), a/L, h/b* and *ln (b/a)*. The experimental results show that the presented method allows a good estimation of pH detection of the material surfaces.

## 1. Introduction

The pH value has a significant impact on the quality and longevity of industrial and cultural heritage materials and objects, paper and packaging materials, forest products, construction materials and others. Therefore, pH is an important measured parameter in many areas, such as materials science and product manufacturing, storage, utilization, degradation, neutralization, mass deacidification, recycling, and evaluation of efficacy and quality of the related devices and technologies. It is also important for testing and quality management systems (QMSs).

When measuring the surface pH of porous materials [1] using pH meters or extraction methods [2,3,4,5], false results can be produced by redistribution of ions in the material structure caused by the application of water used for measurements or extraction. Examples of such false artifacts caused by improper measurement methods are given by several sources [6,7].

These problems can be mitigated by colorimetric pH measurement using suitable pH color indicators impregnated in situ into the measured porous material.

Color and other optical properties, namely those compatible with human vision, including the CIELab color space (three-dimensional color space defined by the International Commission on Illumination (abbreviated CIE)) variables, play an important role in many fields of rapid, low cost and human-like quality evaluation and decision-making, in terms of appearance, environmental quality, habitability, biocompatibility, natural character of materials and longevity assessment, including visual pH evaluation and colorimetric pH measurement [8]. A good example is fiber impregnation with color indicators and their colorimetric measurement for the automatization of objective identification of kinds of fibers [9,10].

The pH indicators incorporated in situ into a fibrous material can be widely used in various applications, like monitoring of food freshness and quality [11], wound dressings able indicate the evolution of the healing process, protective clothing, filtration, etc. [12,13,14].

Measuring the change in pH using color indicators can be used to assess the biological degradation of materials using a biodegradation-specific pH indicator [15], such as pH measurement of the biological damage of test samples. Good examples are spruce wood chips used in pulp and paper, and wood logs in the woodworking and furniture industries stained with the pH-indicator bromophenol [16,17].

To control the effectiveness of paper deacidification, Strlič [18] described a colorimetric method to quantify changes in reflectance spectra to determine the pH values of model samples. Boone used a pH indicator to impregnate a paper sample before deacidification to observe the neutralization effect after treatment [19]. Objective measurement of the 3D distribution of pH across a material’s structure was designed and tested to evaluate the efficacy of porous material deacidification [7,20].

This paper focuses on objective colorimetric pH measurement, based on a combination of impregnation of a tested material with a pH indicator in situ and its exposure to a tested process of technology, colorimetric measurement and data processing. Subsequently, based on regression analysis of CIE orthogonal and cylindrical color variables and derived pH characteristic parameters (pH-CPs), the best pH-CPs are selected. The CIE 1976 (*L, a, b*) (CIELab) color system was used in this paper. The orthogonal *L a b* and/or cylindrical chroma *C* and hue *h* parameters of the CIELab allow for a visualization of the pH and pH-related properties, their distribution and kinetics in the orthogonal system of the 3 axes *L*(lightness), *a* (redness-greenness), *b* (yellowness-blueness).

The colorimetric pH measurement can be used to measure the geometrical outer surfaces’ pH. This information is important for an assessment of materials’ quality and longevity [6,21,22]; quality testing and management systems of materials [23], biomaterials, natural polymer materials and products such as cellulose, wood, paper [24,25,26,27,28,29,30] and building materials [31]; as well as in medical applications, such as the testing of skin [32,33,34].

A similar application of the new pH-characteristic CIELab properties (pH-CPs) can be used in automatization, quality management systems, feedback and feedforward control of modification and production processes of materials.

## 2. Results

This paper describes a rapid, low-cost colorimetric pH and pH distribution measurement of materials. The method using prestained samples can be used in such technological applications and QMSs where Whatman pH indicator paper, paper-printed comparison charts and colorimetric microfluidic paper-based analytical devices are not applicable.

The method consists of the following steps:

1. impregnation of a tested material with pH indicator in situ;
2. its exposure to a tested process of technology;
3. colorimetric pH measurement;
4. generation of new candidate pH characteristic parameters and selection of the best pH-CPs.

**Figure 1:**
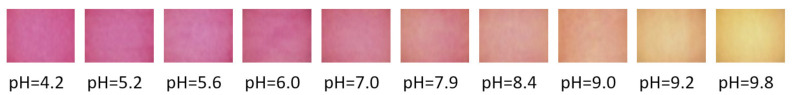
The calibration set of the pH indicator containing samples in the range pH 4–10. Acidic papers were impregnated with the methyl-red solution and were alkalized by magnesium bicarbonate.

An experimental verification of the hypotheses was made using a pH indicator impregnating a model material, in our case acid wood paper, using methyl red (MR) as the model pH indicator. The water deacidification process, used in archival document conservation, was chosen as the tested technology. A calibration sample set with pH in the range 4 to 10 (as measured by a pH meter) was prepared (see Figure 1) and color data in the CIE 1976 Lab color space were obtained.

The first step in the analysis of measured data was a simple linear regression of all measured data—colorimetric parameters *L, a, b, C, h*, their ratios and differences, and the pH value. The most statistically significant results from the simple regression of orthogonal and cylindrical color variables and derived pH-CPs according to the adjusted R squared values are given in Table 1.

The values of adjusted R squared given in Table 1 show that the best fitting models for pH prediction have been obtained with the following pH-CPs, in decreasing order of significance:

*a*/*L* > *h*/*b* >*a* > *a*/*b* > *L* > Δ*a* > Δ*L* > Δ*E* (1976) > Δ*E* (1994) > Δ*E* (CMC 1:1) > Δ*H* > *h*

where the highest value of adjusted R^2^ is 98.68% (*a/L*).

In the next phase, the dependence of pH values on the colorimetric parameters *L, a, b, C, h* was studied. To obtain the best fitting model using several parameters, the backward selection methods were applied.

The most statistically significant results from the regression of orthogonal and cylindrical parameters of pH-CP according to the values of adjusted R squared can be seen in Table 2.

Based on the regression analysis, the order of the most significant color parameters dependent on pH according to the values of adjusted R squared is as follows:

√∆*E* CMC(1:2) > √∆*E* (2000) > √∆*E* CMC(1:1) > √∆*E* (1994) > *ln(a)* > √∆*E* (1976) > √∆*H*(*ab*) > *a*/*L* > *h*/*b* > *ln(b/a)* > *a* > *a*/*b* > *L* > ∆*a* > ∆*L* > ∆*E* (1976) > ∆*E* (1994) > ∆*E* CMC(1:1) > ∆*E* CMC(1:2) > ∆*b*/∆*a* > ∆*h*(*ab*) > ∆*H*(*ab*) > ∆*E* (2000) > ∆*h*.

The analysis of multiple regression shows that the combinations of all selected variables (listed in Table 2) have the highest value of adjusted R^2^. However, the backward selection of variables shows only minor effects of variables other than *a*. Therefore, in this case, the most significant pH-CP variable is *a*.

An example is given for the process analysis of the dependent variable pH and independent variables *L, a, b*; see Equations (1) and (2):

(1) pH=13.4012−0.014403×L−0.197729×a−0.0031366×b

Stepwise regressionMethod: backward selection (MSE—Mean Squared Error)F-to-enter: 4.0, F-to-remove: 4.0Step 0:3 variables in the model.16 d.f. for error.
R^2^ = 97.86%Adjusted R^2^ = 97.46%MSE = 0.106863Step 1:Removing variable *L* with *F*-to-remove = 0.001460022 variables in the model.17 d.f. for error.
R^2^ = 97.86%Adjusted R^2^ = 97.61%MSE = 0.100586Step 2:Removing variable *b* with *F*-to-remove = 0.003548341 variable in the model.18 d.f. for error.
R^2^ = 97.86%Adjusted R^2^ = 97.74%MSE = 0.0950179

(2) pH=11.9918−0.186781×a

## 3. Discussion

The regression shows a strong dependence between all types of total color difference and the pH value. In this case, it is not important which formula for the total color difference is used. In all cases, Δ*E* is the total color difference for the orthogonal parameters *L, a, b,* between the measured sample and the reference, with the most acidic paper standard site used with pH = 4.2; *L_ref_* = 64.4, *a_ref_* = 41.3, *b_ref_* = 10.8.

∆*H_ab_* and ∆*h_ab_* are differences in cylindrical parameters *L, C, H_ab_* between the measured sample and the reference, with the most acidic paper standard site used with pH = 4.2; *L_ref_* = 64.4, *C_ref_* = 42.7, *H_ab ref_* = 14.5°.

The reason for this is the fact that during the transition from the acidic to the alkaline environment, there is a transition from red to yellow and within this transition, there is a shift to lower values of the coordinate *a* as well as an increase in the value of *b*, and there is also a small difference in *L* values.

Multiple regression analyses carried out reveal the major influence of the value of orthogonal coordinates *a, b* and cylindrical coordinates *C, H_ab_*. The best pH-CPs with the highest correlation parameters with pH are listed in Table 3.

## 4. Materials and Methods

### 4.1. Materials

Test acid lignin-containing paper was used from Klug-Conservation (Immenstadt im Allgäu, Germany). Methyl red (MR) was purchased from Sigma-Aldrich (Darmstadt, Germany). Magnesium hydroxide was supplied by Sigma-Aldrich.

### 4.2. Methods

#### 4.2.1. Sample Preparation

The indicator was dissolved (1 g/L MR ethanol:water/2:1). Test paper was cut to size 5 × 5 cm. The samples were immersed in indicator solution for 1 min. After dyeing, all samples were dried under laboratory conditions.

The alkali solution of magnesium bicarbonate was prepared by adding 12 g/L of magnesium hydroxide into a pressure reactor and mixing under a pressure of 5 atm for 2 h in a CO_2_ atmosphere. The set of fresh calibration solutions was prepared from 0.2 to 0.0002 mol/L (temperature conditions 4 °C).

**Figure 2:**
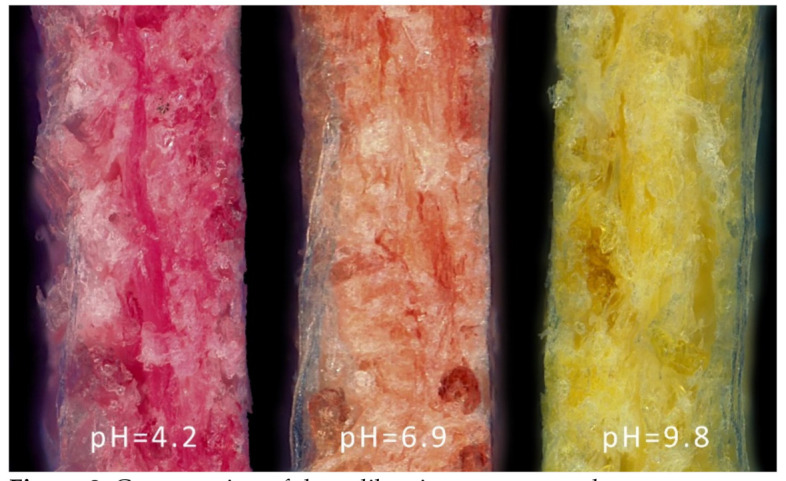
Cross-section of the calibration paper samples.

The calibration samples were prepared by immersing the dyed samples into alkali solutions for 1 min. Subsequently, the samples were air-dried under laboratory conditions. The homogeneity of indicator staining, as well as the homogeneity of neutralization, were observed in the cross-section of the samples by optical microscopy (transverse and longitudinal section of the paper, Figure 2).

#### 4.2.2. pH Measurement

The pH meter Jenway 3510 (Bibby Scientific Ltd, Staffordshire, UK) was used. Surface pH measurements were executed with a flat-surface combined pH electrode (Mettler TOLEDO, Columbus, OH, USA). According to the Tappi 529 [1] standard method, a drop of water (50 μL) was placed on a sample, the flat-surface combined pH electrode pressed against it, and the pH value read after being constant for 30 s. The results are an average of 10 determinations.

#### 4.2.3. Color Parameter Measurement

The coordinates were measured with a Spectro-Densitometer (SpectroDens, Techkon, GmbH, Königstein, Germany) by CIE-Lab using a D50/2° illuminant without polarization, according to the Tappi 524 om-94 [35]. The samples were measured on 5 different spots on both sides. The average value of the measurement was calculated from two parallels. *L, C, h* coordinates were calculated from *L, a, b* coordinates (Equations (3) and (4)):

(3) $$C_{ab}^{}=\sqrt{a^{2}+b^{2}}$$

(4) $$h_{ab}=\tan^{-1}\left(\frac{b^{}}{a^{}}\right)$$

*L* represents lightness, *a* is approximate redness-greenness, *b* is approximate yellowness-blueness, *C_ab_* is chroma, and *h_ab_* is hue. The *L*, *a* and *b* coordinates are used to construct a Cartesian color space. The *L*, *C_ab,_* and *h_ab_* coordinates are the cylindrical representation of the same space.

Color differences are measured in the CIELab space as the Euclidean distance between the coordinates for the two stimuli. This is expressed in terms of a CIELab Δ*E_ab_*, which can be calculated using Equation (5):

(5) $$\Delta E_{ab}^{}=\sqrt{\Delta L^{2}+\Delta a^{2}+\Delta b^{2}}$$

The differences between the reference sample (the most acidic sample) and the sample from the calibration set were compared. The magnitude of the color difference was quantified by Δ*E_ab_*, with *ΔL* the lightness difference, and Δ*a* and Δ*b* the difference in *a* and *b* values.

It can also be expressed in terms of lightness, chroma (Δ*C_ab_*), and hue (Δ*H_ab_*) differences as illustrated in Equations (6) and (7):

(6) $$\Delta E_{ab}^{}=\sqrt{\Delta L^{2}+\Delta C_{ab}^{2}+\Delta H_{ab}^{2}}$$

(7) $$\Delta H_{ab}^{}=\sqrt{\Delta E_{ab}^{}-\Delta L^{2}-\Delta C_{ab}^{2}}$$

While the CIELab color space was designed to have color differences perceptually uniform throughout the space, this goal was not strictly achieved.

To improve the uniformity of color difference measurements, modifications to the CIELab Δ*E_ab_* equation have been made based upon various empirical data. One of the most widely used modifications is the CMC color difference equation [36], which is based on a visual experiment on color difference perception in textiles. The CIE (1995b) has recently evaluated such equations and the available visual data, recommending a new color difference equation for industrial use. This system for color difference measurement is called the CIE 1994 (Δ*L*, Δ*C_ab_*, Δ*H_ab_*) color difference model.

The last difference that the CIE established was the CIE DE2000 color difference equation [37] that extends the concept of CIE94 with further complexity.

While the statistical validity of the DE2000 computations for most real-world applications is questionable, the equation is gaining in popularity and most certainly represents an improvement over the simple CIELab color difference equation for almost any application [38].

Statistical data processing was performed by methods of regression analysis. The results of fitting several curvilinear models to the data were found and compared by the R^2^ values. Since the models with an unequal number of parameters were compared, to obtain a significant comparison, the values of R squared were adjusted. All measured data were processed in the program Statgraphics Centurion XV, Version 15.2.05.

## 5. Conclusions

The key idea and aim of this work was to generate and test candidate pH characteristic parameters for a quick, low-cost, rapid measurement of pH. The procedure used consists of impregnation of a tested material with pH indicator in situ, further of its exposure to a tested process of technology (colorimetric pH measurement) then of correlation analysis of CIE orthogonal and cylindrical color variables and derived pH characteristic parameters (pH-CP), and finally of selecting the best pH-CP.

A simple linear regression of all measured data was performed. The most statistically significant results among orthogonal and cylindrical color variables and derived pH-CP variables according to the adjusted R^2^ were *a/L* (98.68%), *h/b* (98.03%), *a* (97.92%) and *a/b* (97.83%).

The color CIE parameters, obtained by regression of orthogonal and cylindrical parameters of pH-CPs according to the values of adjusted R^2^, best correlating with pH, and therefore the most suitable for measurement of pH of used materials, are as follows: *√*∆*E* CMC(1:2), *√*∆*E* (2000), *√*∆*E* CMC(1:1), *√*∆*E* (1994) (99.11%–98.97%), *ln(a)* (98.91%), *√*∆*E*(1976) (98.84%), *√*∆*H*(ab) (98.78%), *a/L* (98.68%), *h/b* (98.03%) and *ln(b/a)* (98.01%). The backward selection of variables shows that, in this case, the most significant pH-CP variable and thus the most characteristic value for pH determination in the case of the paper substrate is the coordinate *a*.

The experimental results obtained using the pH-indicator impregnation in combination with the best pH-CP show that it allows for a good estimation of pH values of the external surfaces. Superior and easy pH estimation is achieved using colorimetric pH-CP = *√*∆*E* measurement in the application simply using surface CIELab color data for the material impregnated with color pH indicator. The application of the above-mentioned method seems to be promising for the evaluation of pH values of paper microstructures [7].

## 6. Patents

Katuščák, S., Vodný, Š., Vizárová, K.: pH Distribution measurement in a porous material microstructure. Method and apparatus. PCT/IB2018/053927, WO/2019/229504 (2019).

## Figures and Tables

**Table 1:** The best results for the simple linear regression between pH-CPs and pH.

**pH = 12.0005 − 0.187229 × *a***	**pH = −16.3868 + 0.318285 × *L***	**pH = 3.66558 + 0.0896278 × *h***
R2 (adjusted for d.f.) = 97.92%	R2 (adjusted for d.f.) = 97.81%	R2 (adjusted for d.f.) = 94.13%
**pH = 11.2954 − 11.3262 × *a*/*L***	**pH = − 4.38203 + 6.21752 × *h*/*b***	**pH = 9.83382 − 1.34982 × *a*/*b***
R2 (adjusted for d.f.) = 98.68%	R2 (adjusted for d.f.) = 98.03%	R2 (adjusted for d.f.) = 97.83%
**pH = 4.27236 − 0.186781 × Δ*a***	**pH = 4.09775 + 0.319191 × Δ*L***	**pH = 5.03303 + 0.139455 × Δ*H***
R2 (adjusted for d.f.) = 97.74%	R2 (adjusted for d.f.) = 97.63%	R2 (adjusted for d.f.) = 94.58%
**pH = 4.35687 + 0.1365 × Δ*E* (1976)**	**pH = 4.46478 + 0.183391 × ∆*E* (1994)**	**pH = 4.5011 + 0.192817 × Δ*E* (CMC 1:1)**
R2 (adjusted for d.f.) = 97.41%	R2 (adjusted for d.f.) = 96.75%	R2 (adjusted for d.f.) = 96.58%

d.f. — means the degrees of freedom. Adjusted R^2^ is a modified version of R-squared that has been adjusted for the number of predictors in the model.

**Table 2:** Determination of pH materials, statistically significant expressions. The values of adjusted R^2^ for relationships between candidate surface pH characteristic CIE color parameters (the tested orthogonal and cylindrical parameters, derived ratios, and total and partial color differences including both differences of hue Δ*h_ab_* and Δ*H_ab_*) and the surface pH of material (model paper samples—methyl red indicator containing acid paper with pH_0_ = 4.2); modified using Mg(HCO_3_)_2_ solution gradually diluted c mol/L <0.0002, 0.2>; surface pH values of samples <4.3, 9.8>).

Characteristic Parameter; pH-CP	Correlation Coefficient; r	R^2^ %	R^2^ (Adjusted for d.f.) %	Equation
Simple Regression				
√∆*E* CMC (1:2)	0.9958	99.15	99.11	pH = 2.98229 + 1.29486 × √∆*E*
√∆*E* (2000)	0.9956	99.13	99.08	pH = √(3.35706 + 16.7181 × √∆*E*)
√∆*E* CMC (1:1)	0.9954	99.08	99.03	pH = 2.83735 + 1.28324 × √∆*E*
√∆*E* (1994)	0.9951	99.02	98.97	pH = 2.76226 + 1.26054 × √∆*E*
*ln(a)*	−0.9948	98.97	98.91	pH = √(162.231 − 38.3235 × *ln(a))*
√∆*E* (1976)	0.9945	98.90	98.84	pH = 2.57385 + 1.10343 × √∆*E*
√∆*H*(*ab*)	0.9942	98.84	98.78	pH = 4.39773 + 0.874399 × √∆*H*
*ln(b/a)*	0.9905	98.11	98.01	pH = √(64.3279 + 29.7346 × *ln(b/a))*
*a*	−0.9896	97.93	97.92	pH = 12.005 − 0.187229 × *a*
∆*E* (1976)	0.9877	97.55	97.41	pH = 4.35687 + 0.1365 × ∆*E*
∆*E* (1994)	0.9845	96.92	96.75	pH = 4.46478 + 0.183391 × ∆*E*
∆*E* CMC (1:2)	0.9826	96.55	96.36	pH = 4.56554 + 0.202441 × ∆*E*
∆*b*/∆*a*	−0.9802	96.08	95.86	pH = (2.38378 − 0.920062 × ∆*b*/∆*a*)^2^
∆*b*/∆*a*	0.9794	95.92	95.70	pH = 5.78191 − 4.78967 × ∆*b*/∆*a*
∆*H*(*ab*)	0.9754	95.14	94.87	pH = √(26.6656 + 1.3561 × ∆h)
∆*H*(*ab*)	0.9740	94.86	94.58	pH = 5.03303 + 0.139445 × ∆*H*
∆*E* (2000)	0.9668	93.47	93.11	pH = 4.82897 + 0.185748 × ∆*E*
∆*H*	0.9570	91.58	91.12	pH = 5.15388 + 0.0948435 × ∆*h*
∆*C*	0.9192	84.49	83.62	pH = 1/(0.247154 + 0.0120777 × ∆*C*)
∆*C*	−0.8543	72.99	71.49	pH = 3.34293 − 0.482664 × ∆*C*
Multiple Regression				
*a*, *b*, ∆*E*, *b*/*a*, *L*/*b*		99.52	99.35	pH = 13.9907 − 0.564697 × *a* + 0.689466 × *b* − 0.923511 × ∆*E* + 1.41696 × *b*/*a* + 0.948398 × *L*/*b*
*H*/*L*, *H*/*C*, *C*/*L*		99.49	99.39	pH = 10.3343 + 22.3283 × *h*/*L* − 6.80628 × h/*C* − 13.4651 × *C*/*L*
∆*H*, ∆*H*(*ab*), ∆*C*		99.17	99.01	pH = 4.26073 + 0.165637 × ∆*H* − 0.0378499 × ∆*h* − 0.152348 × ∆*C*
*L*, *C*, *H*		99.06	98.88	pH = 16.5441 − 0.00774926 × *L* − 0.199133 × *C* + 0.0860289 × *h*
*C*, *H*		99.03	98.91	pH = 10.5355 − 0.171271 × *C* + 0.0678806 × *h*
*a*, *L*/*a*		99.01	98.90	pH = 14.5622 − 0.242712 × *a* − 0.302836 × *L*/*a*
√∆*H*, ∆*H*		98.87	98.66	pH = 1.40643 + 0.921009 × √∆*H* + √(8.74598 + −0.0307949 × ∆*h*)
*b*/*a*, ∆*E*(1976)		98.98	98.86	pH = 4.25415 − 0.747491 × *b*/*a* + 0.182809 × ∆*E*
∆*b*/∆*a*, ∆*H*		98.07	97.84	pH = 5.40496 − 2.70015 × ∆*b*/∆*a* + 0.0646712 × ∆*H*
*L*, *a*, *b*		97.86	97.46	pH = 13.4012 − 0.014403 × *L* − 0.197729 × *a* − 0.0031366 × *b*

**Table 3:** The most significant relationships between pH and orthogonal (a) and cylindrical (b) CIELAB parameters according to the adjusted R^2^ values.

(a)	(b)
*L, a, b*	*L, C, h*
pH = √(162.231 − 38.3235 × *Ln*(*a*)) R^2^ = 98.91%	pH = 10.3343 + 22.3283 × *h*/*L* − 6.80628 × *h*/*C* − 13.4651 × *C*/*L* R^2^ = 99.39%
pH = 14.5622 − 0.242712 × *a* − 0.302836 × *L*/*a* R^2^ = 98.90%	pH = 16.5441 − 0.00774926 × *L* − 0.199133 × *C* + 0.0860289 × *h* R^2^ = 98.88%
pH = 11.2954 − 11.3262 × *a*/*L* R^2^ = 98.68%	pH = 10.5355 − 0.171271 × *C* + 0.0678806 × *h* R^2^ = 98.91%

## Data Availability

The data presented in this study are available on request from the corresponding author. The data are not publicly available due to the fact that research is still in progress.

## Sample Availability

Samples of the test papers are available from the authors upon request.
